# High incidence and persistence of hepatitis B virus infection in individuals receiving HIV care in KwaZulu-Natal, South Africa

**DOI:** 10.1186/s12879-020-05575-6

**Published:** 2020-11-16

**Authors:** Nokukhanya Msomi, Kogieleum Naidoo, Nonhlanhla Yende-Zuma, Nesri Padayatchi, Kerusha Govender, Jerome Amir Singh, Salim Abdool-Karim, Quarraisha Abdool-Karim, Koleka Mlisana

**Affiliations:** 1 Discipline of Virology-University of KwaZulu-Natal, School of Laboratory Medicine and Medical Sciences and National Health Laboratory Service, Durban, South Africa; 2 Centre for the AIDS Programme of Research in South Africa, University of KwaZulu-Natal, Durban, South Africa; 3 MRC-CAPRISA HIV-TB Pathogenesis and Treatment Research Unit, Doris Duke Medical Research Institute, University of KwaZulu-Natal, Durban, South Africa; 4 Dalla Lana School of Public Health, University of Toronto, Toronto, Canada; 5 Department of Epidemiology, Mailman School of Public Health, Columbia University, New York, USA; 6 National Health Laboratory Service and School of Laboratory Medicine and Medical Sciences, University of KwaZulu-Natal, Durban, South Africa

**Keywords:** HBV incidence, HIV/HBV coinfection, South Africa

## Abstract

**Background:**

Hepatitis B virus (HBV), Human Immunodeficiency virus (HIV) and Tuberculosis (TB) are common infections in South Africa. We utilized the opportunity of care provision for HIV-TB co-infected patients to better understand the relationship between these coinfections, determine the magnitude of the problem, and identify risk factors for HBV infection in HIV infected patients with and without TB in KwaZulu-Natal, South Africa.

**Methods:**

This retrospective cohort analysis was undertaken in 2018. In-care HIV infected patients were included in the analysis. Results from clinical records were analysed to determine the prevalence, incidence, persistence and factors associated with HBsAg positivity in HIV-infected patients with or without TB co-infection.

**Results:**

A total of 4292 HIV-infected patients with a mean age of 34.7 years (SD: 8.8) were included. Based on HBsAg positivity, the prevalence of HBV was 8.5% (363/4292) [95% confidence interval (CI): 7.7–9.3] at baseline and 9.4% (95%CI: 8.6–10.3%) at end of follow-up. The HBV incidence rate was 2.1/100 person-years (p-y). Risk of incident HBV infection was two-fold higher among male patients (HR 2.11; 95% CI: 1.14–3.92), while severe immunosuppression was associated with a greater than two-fold higher risk of persistent infection (adjusted risk ratio (RR) 2.54; 95% CI 1.06–6.14; *p* = 0.004. Additionally, active TB at enrolment was associated with a two-fold higher risk of incident HBV infection (aHR 2.38; 95% CI: 0.77–7.35).

**Conclusion:**

The provision of HIV care and treatment in high HBV burden settings provide a missed opportunity for HBV screening, immunization and care provision.

## Background

Human Immunodeficiency Virus (HIV) and Hepatitis B Virus (HBV) infections remain two of the major healthcare challenges in the twenty-first century. It is estimated that there were 257 million persons living with chronic HBV infection globally (WHO 2017 global hepatitis report), making it the most common chronic viral infection worldwide [1]. In six WHO regions, global HBV surface antigen (HBsAg) sero-prevalence is reported to be 3,61% (95% CI 3·61–3·61) in the general population, with most countries in Africa shown to have higher-intermediate endemicity (HBsAg prevalence 5–7·99%), or highly endemic for HBV (HBsAg prevalence ≥8%) [2]. Among the estimated 40 million people living with HIV globally, about 10% are concurrently chronically infected with HBV [3]. In sub-Saharan Africa, there is a syndemic of HIV, HBV and tuberculosis (TB). A syndemic is defined as a clustering of two or more disease epidemics that interact with each other increasing the magnitude of each epidemic [4].

The clinical disease spectrum of HIV, TB and HBV co-infection include hepatitis, either due to direct pathogen insult to the liver or as a complication of hepatotoxic drugs used to treat these infections. Some antiretroviral drugs commonly used in the treatment of HIV such as Tenofovir and Lamivudine, are also effectively used in the treatment of HBV. The pro-inflammatory state of chronic HIV infection drives the replication and evolution of pathogens [5] hence, upon HIV diagnosis, current standard of care recommends investigations to exclude commonly occurring infections like viral hepatitis and tuberculosis, especially in countries where these syndemics are prevalent. This also ensures that co-morbid infections are properly diagnosed and treated.

In HBV endemic areas like sub-Saharan Africa, most HBV infections occur perinatally and in early childhood, and both infections are more likely to progress to chronicity [6] in the absence of immunization. Amongst adults, HBV infection typically presents as acute hepatitis and progression to chronic HBV is estimated to be < 1% in immunocompetent individuals [7]. There is paucity of data on rates of incident HBV infection in adults. Furthermore, data on rates of persistence and/or clearance of HBV in the presence of HIV infection also remains scarce. Data from outside Africa indicate that the natural course of HBV infection is altered in the presence of HIV infection, showing higher levels of HBV viraemia, frequent episodes of reactivation and more rapid progression of liver fibrosis and hepatocellular carcinoma [8–10]. The EuroSida Swiss cohort study reported a significantly higher incidence of all-cause and liver-related mortalities in HBsAg-positive participants at 3.7 and 0.7/100 person-years respectively, compared with HBsAg-negative participants (2.6/100 and 0.2/100 person-years, respectively). Liver-related mortalities were three fold higher in HBsAg-positive subjects at 0.7/100 person-years compared with HBsAg-negative subjects at 0.2/100 person-years [11]. A nationwide study in Japan, a country with high HBV endemicity investigated risk factors for long term persistence of HBV in adults and concluded that genotype A is an independent risk factor for long term persistence of HBV [12]. Notably, genotype A has been shown to be the predominant circulating genotype in Africa [13]. Subic et al. highlight the barriers to the diagnosis and adequate treatment of HBV with estimation that only 10% of the 257 million people living with HBV have been diagnosed, and as few as 1% are being adequately treated [14].

Quantifying the burden and nature of HBV among TB-HIV co-infected South African patients accessing treatment and care services, is an important first step to enhance management of associated clinical complexities arising in the care of affected patients.

We conducted a retrospective chart review to assess the prevalence, incidence and persistence of HBV infection in HIV infected individuals accessing antiretroviral therapy in KZN, South Africa.

## Methods

### Study design

We undertook a retrospective chart review of HIV infected ART naïve adult patients enrolled in the PEPFAR funded CAPRISA AIDS Treatment Program (CAT) [15], and patients enrolled in the Starting Antiretroviral Therapy at Three Points in Tuberculosis (SAPiT) trial [16]. We analysed routine hepatitis B surface antigen (HBsAg) serology and other clinical data to determine baseline and overall prevalence of HBV during the follow up period, the number of new infections; the proportion of patients clearing infection or remaining with persisting HBV infection while being managed for HIV with or without tuberculosis.

#### CAT cohort

Patients in the CAT program were enrolled from two catchment populations in KwaZulu-Natal; a TB clinic in the urban eThekwini district of Durban and a rural primary health care clinic in the Vulindlela district. Patients were initiated on antiretroviral therapy (ART) in accordance with the 2004 eligibility criteria of the South African Government HIV/AIDS treatment guidelines [17]. Following ART initiation, clinical and adherence assessments were undertaken weekly for the first 2 weeks, then monthly for the first 6 months and every 3 months thereafter unless clinically indicated. Laboratory safety assessments, CD4+ cell count, and viral loads were conducted at baseline and every 6 months or as clinically indicated. The laboratory safety assessments included liver function tests and screening for HBV at baseline and 6-month intervals using Hepatitis B surface antigen (HBsAg) testing. Patients were regarded as lost to follow up (LTFU) if they missed three consecutive scheduled visits. No LTFU patients were re-enrolled.

#### SAPiT cohort

The SAPiT trial was conducted at the urban Centre for the AIDS Programme of Research (CAPRISA) eThekwini clinic. The CAPRISA eThekwini clinic adjoins the Prince Cyril Zulu Communicable Disease Centre, which is an outpatient TB facility. This was a 3-arm randomized open-label clinical trial in 642 patients where the primary outcome of the trial was to determine the optimal timing of ART initiation in patients co-infected with HIV and TB. The study concluded that the initiation of antiretroviral therapy during tuberculosis therapy significantly improved survival and provides further impetus for the integration of tuberculosis and HIV services [18]. A secondary objective of this study was to compare IRIS risks and outcomes in patients initiating ART within a month of TB treatment initiation [19]. Laboratory safety assessments included screening for HBV at 6 months intervals.

### Laboratory methods

HBsAg was used to screen for HBV as part of laboratory safety assessments at baseline and repeated at 6-month intervals. The HBsAg tests were done on the Architect analyzer (Abbott Laboratories, Wiesbaden-Germany) from 2004 to 2010 and the ADVIA Centaur system (Siemens Healthcare Diagnostics, Tarrytown-NY USA) from 2011 to 2013 as per manufacturers’ instructions. These immunoassays are registered for in vitro diagnostic use. HIV screening and confirmatory diagnosis was done by two rapid HIV tests and TB diagnosis was based on sputum smear positive for acid fast bacilli (AFB) by auramine and Ziel-Niehlsen stains. The TB and HIV diagnostic tests used have been previously described [18].

### Statistical analysis

Wilcoxon rank sum test and Fisher’s exact test were used to compute the association between gender, prevalent HBsAg positivity and other continuous and categorical baseline characteristics. The incidence of HBV and duration of follow-up among patients who tested HBsAg positive was calculated as time from the first HBsAg negative test date to the midpoint of last negative and first positive HBsAg test date. For patients who remained HBsAg negative, duration of follow-up was calculated as time from baseline HBsAg negative date to the last negative date. Univariable and multivariable proportional hazards regression was used to assess predictors of incident HBV infection. The Kaplan Meier method was used to calculate cumulative probability of HBV infection. Moreover, Poisson regression with robust variance was used to assess factors associated with HBV persistence. The following baseline variables were tested: age, gender TB comorbidity, the level of immunosuppression as determined by CD4+ count and body mass index. Statistical analyses were conducted using SAS version 9.4 (SAS Institute, Cary, North Carolina).

### Definitions for HBV prevalence, incidence, clearance and persistence

The World Health Organization (WHO) guidelines for the prevention, care and treatment of persons with chronic HBV infection recommend that high-risk groups be screened for HBV infection by the detection of HBsAg. Chronic HBV infection is defined as persistence of HBsAg for more than 6 months.

We defined overall prevalence as the total number of patients with a positive HBsAg result at baseline and or during follow-up among all those tested. Each positive patient result was counted once.

Cumulative incidence was defined as the proportion of new HBsAg positive cases throughout the follow up period and this include cases with re-infection. The incidence rate was calculated per 100 person years.

Clearance was defined as a loss of HBsAg in those who were previously HBsAg positive and were followed up.

Persistence was defined as those patients whose result remained HBsAg positive with subsequent testing with no evidence of clearance.

During this study, ancillary care related to HBV infection and other diseases besides HIV or TB were managed according to prevailing South African standard clinical practice at the time (2005–2012), which did not include adult HBV immunization. Participants with symptomatic HBV disease were referred to public health sector facilities for further investigation and clinical management.

## Results

### Patient baseline characteristics

Four thousand two hundred and ninety two (4292) HIV infected patients were included in the analyses, of which 2681 (62.5%) were women and 1611 (37.5%) were men. Data on age, gender, clinical status are presented in Table 1. There were statistically significant differences observed in all variables including age, TB status, body mass index, CD4 count and mean HIV viral load at enrolment along gender lines.

**Table 1:** Baseline clinical and demographic characteristics by gender

Variable	Women (***N*** = 2681)	Men (***N*** = 1611)	***p***-value
Research site, n (%)			< 0.001
Urban	1271 (47.4%)	973 (60.4%)	
Rural	1410 (52.6%)	638 (39.6%)	
Age group (years), n (%)^a^			< 0.001
< 24	227 (8.5%)	48 (3.0%)	
24–34	1412 (52.8%)	701 (43.6%)	
≥ 35	1036 (38.7%)	859 (53.4%)	
TB status at enrollment, n (%)			< 0.001
TB not present	1910 (71.2%)	904 (56.1%)	
TB present	771 (28.8%)	707 (43.9%)	
Body mass index (kg/m^2^), n (%)^b^			< 0.001
< 18.5	227 (8.8%)	301 (19.3%)	
≥ 18.5	2361 (91.2%)	1260 (80.7%)	
CD4 count at enrollment (cells/mm^3^), n(%)^c^			< 0.001
< 50	363 (15.2%)	336 (22.9%)	
50–200	1236 (51.9%)	777 (52.9%)	
≥ 200	782 (32.8%)	356 (24.2%)	
Viral load (log_10_ copies/ml), mean ± SD	4.9 ± 0.9	5.1 ± 0.8	< 0.001

^a^ 9 patients had missing data

^b^ 143 patients had missing data

^c^ 442 patients had missing data

### HBV prevalence

A total of 363 patients [8.5, 95% confidence interval (CI): 7.7 to 9.3] were infected with HBV at baseline as determined by HBsAg seropositivity (Table 2). The proportion of patients from urban sites was 52.3% compared to 47.7% who were from rural settings. There was a higher baseline prevalence of HBV in urban sites compared with rural sites (9.6% vs 7.2%; RR: 95% CI; *p* = 0.004). The proportion of HBV positivity at baseline was higher in men compared with women (11.9% vs 6.4% *p* < 0.001). A higher proportion of patients with a low BMI (< 18.5) were HBV positive at baseline compared with patients with a higher BMI (11.6% vs 8.0% *p* = 0.007).

**Table 2:** Baseline Clinical and Demographic Characteristics by HBsAg Status

Variable	HBsAg Positive (***N*** = 363)	HBsAg Negative (***N*** = 3929)	***p***-value
Research site, n (%)			0.004
Urban	216 (9.6%)	2028 (90.4%)	
Rural	147 (7.2%)	1901 (92.8%)	
Gender, n (%)			<.001
Male	192 (11.9%)	1419 (88.1%)	
Female	171 (6.4%)	2510 (93.6%)	
Age group (years), n (%)^a^			0.403
< 24	22 (8.0%)	253 (92.0%)	
24–34	191 (9.0%)	1922 (91.0%)	
≥ 35	149 (7.9%)	1746 (92.1%)	
TB status at enrollment, n (%)			0.564
TB not present	233 (8.3%)	2581 (91.7%)	
TB present	130 (8.8%)	1348 (91.2%)	
Body mass index (kg/m^2^), n (%)^b^			0.007
< 18.5	61 (11.6%)	467 (88.4%)	
≥ 18.5	288 (8.0%)	3333 (92.0%)	
CD4+ count at enrollment (cells/mm^3^), n (%)^c^			0.185
< 50	74 (10.6%)	625 (89.4%)	
50–200	173 (8.6%)	1840 (91.4%)	
≥ 200	93 (8.2%)	1045 (91.8%)	
HIV Viral load (log_10_ copies/ml), mean (SD)	5.0 (0.9)	5.0 (0.9)	0.179

^a^ 9 patients had missing data

^b^ 143 patients had missing data

^c^ 453 patients had missing data

The presence of tuberculosis at enrolment, level of immunosuppression and baseline mean HIV viral load were not associated with HBsAg seropositivity at baseline (Table 2).

**Fig. 1:**
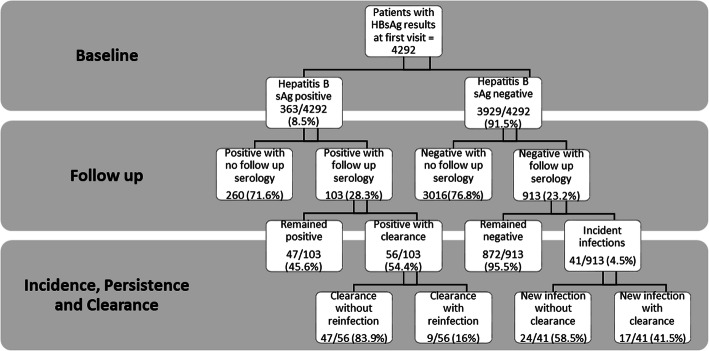
Flowchart depicting prevalence, incidence, clearance and persistence of HBV among HIV infected patients

Data on incidence rates, persistence and clearance in relation to baseline prevalence are presented in Fig. 1 flowchart. A total of 1016 of the 4292 patients (23.7%) had subsequent HBsAg results. These patients had follow-up results for up to 5 years and were included in the analysis for incidence, clearance and persistence as outlined in Fig. 1. The overall prevalence of HBV at the end of the follow up period was 9.4% (95% CI: 8.6 to 10.3%).

### HBV incidence rates

Data on incident HBV infections are presented in Table 3. Among the 913 patients who were HBsAg negative at baseline with follow-up results, we identified 41 incident HBV infections occurring over 1920 person years (p-y) yielding an overall HBV incidence rate of 2.1/100 p-y. Male gender was associated with a two-fold higher risk of having incident HBV infection [HR 2.11; 95% CI: 1.14–3.92 *p* = 0.017], while not statistically significant in the multivariable analyses, [aHR 1.72; 95% CI 0.89–3.35 *p* = 0.108] this may be of clinical relevance. Other factors associated with incident HBV infection including patients from a rural site and patients with TB at baseline are summarized in Table 3.

**Table 3:** Factors associated with incident HBV infection

Baseline characteristics	HR (95% CI)	***p***-value	aHR (95% CI)	***p***-value
Gender (ref: female)				
Male	2.11 (1.14–3.92)	0.017	1.72 (0.89–3.35)	0.108
Research site (ref: urban)				
Rural	1.14 (0.50–2.59)	0.758	3.04 (0.82–11.21)	0.095
Age group (years) (ref: ≥35)				
< 24	1.12 (0.33–3.83)	0.853	1.01 (0.23–4.50)	0.985
24–34	1.13 (0.59–2.13)	0.716	1.20 (0.62–2.33)	0.593
CD4 count (cells/mm^3^) (ref: ≥200)				
< 50	1.35 (0.53–3.39)	0.528	1.33 (0.52–3.39)	0.550
50–200	1.09 (0.50–2.39)	0.832	1.18 (0.53–2.63)	0.693
Active TB at enrolment (ref: no)				
Yes	1.57 (0.79–3.13)	0.203	2.38 (0.77–7.35)	0.132
BMI (kg/m^2^) (ref: < 18.5)				
≥ 18.5	0.84 (0.35–1.99)	0.687	1.21 (0.46–3.16)	0.703

**Fig. 2:**
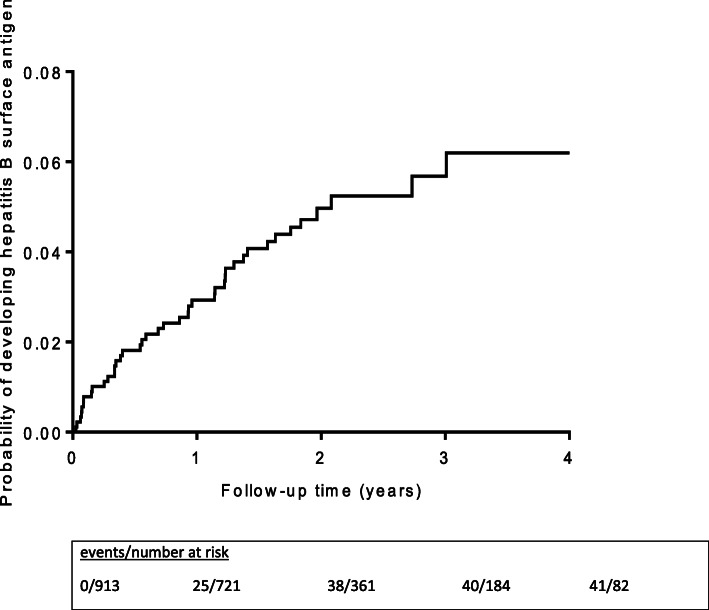
Kaplan-Meier estimates of cumulative probability of incident hepatitis B infection

Despite the small numbers of patients tested over time, and the reduced numbers in follow-up, the Kaplan-Meier curve in Fig. 2, demonstrates a steady cumulative increase in the probability of incident HBV infection over the first 2 years of follow-up.

### Clearance

Of the 41 incident HBV infections, 17(41.5%) subsequently cleared the infection whereas 24 (58.5%) did not have any evidence of clearance as shown by presence of HBsAg.

Among the 56/103 (54.4%) patients who cleared HBV infection during follow-up (95% CI: 44.8 to 63.7), nine (16%) were re-infected and 47(83.9%) did not demonstrate evidence of reinfection at last follow-up visit.

### Persistence

Data on persistent HBV infection are presented in Table 4. There were 103 patients who were HBsAg positive at baseline with follow-up results and 47 (45.6%; 95% CI: 36.3 to 55.2) had persistent HBsAg positivity during follow-up. Predictors of persistent infection included severe immunosuppression where CD4 cell count below 50 cells/uL was associated with a higher risk of developing persistent infection compared to CD4 cell count > 200 cells/uL [adjusted risk ratio (RR) 2.54; 95% CI 1.06–6.14; *p* = 0.004].

**Table 4:** Factors associated with persistent HBV infection

Baseline characteristics	RR (95% CI)	***p***-value	aRR (95% CI)	***p***-value
Gender (ref: female)				
Male	1.75 (1.00–3.06)	0.05	1.71 (0.97–3.02)	0.064
Research site (ref: urban)				
Rural	0.59 (0.24–1.47)	0.254	0.51 (0.17–1.52)	0.226
Age group (years) (ref: ≥35)				
< 24	1.46 (0.51–4.20)	0.478	1.57 (0.47–5.23)	0.463
24–34	1.24 (0.69–2.25)	0.469	1.43 (0.79–2.61)	0.241
CD4 count (cells/mm^3^) (ref: ≥200)				
< 50	2.63 (1.10–6.32)	0.030	2.54 (1.06–6.14)	0.038
50–200	1.57 (0.68–3.58)	0.289	1.54 (0.65–3.63)	0.327
Active TB at enrolment (ref: no)				
Yes	1.17 (0.66–2.10)	0.588	0.80 (0.42–1.52)	0.498
BMI (kg/m^2^) (ref: < 18.5)				
≥ 18.5	1.15 (0.46–2.85)	0.764	1.33 (0.54–3.28)	0.538

## Discussion

We report alarmingly high prevalence rates of HBV infection based on HBsAg positivity in HIV infected patients seeking treatment and care services in South Africa. While these rates are consistent with data reported in other settings in sub-Saharan Africa, this confirms that South Africa remains endemic for HBV, HIV as well as tuberculosis [20]. Notwithstanding higher rates of ARV treatment initiation rates in women compared to men in our cohorts, baseline HBV prevalence was almost two times higher in men compared to women (11.9% vs 6.4%), consistent with findings from settings where heterosexual transmission is dominant. The high HBV prevalence rates among men in our general HIV population is comparable to HBV prevalence rates observed among high risk men having sex with men (MSM) populations where HBV prevalence rates of > 8 and 11% were observed in China and the United States of America [21] [22], respectively. The baseline prevalence of HBV was similar in all age groups, suggesting that our study patients did not benefit from the routine immunization against HBV that was rolled out to infants and newborns in South Africa in 1995.

The high HBV incidence rates in our setting is also deeply concerning. Incidence rates in our cohorts are about four fold higher than rates observed among ART accessing populations in Uganda (0.49/100 p-y [23] vs 2.1/100 p-y), despite more than 90% of our patients receiving either lamivudine or a combination of Tenofovir and lamivudine first line ART regimens. This suggests there is ongoing transmission and acquisition of HBV in ART treated adults and should prompt future surveillance studies on HBV drug resistance in the South African setting.

The high cumulative incidence and cumulative probability of incident HBV infection underscores the missed opportunity of identifying patients susceptible to HBV and the provision of early vaccination to prevent new infections. Similarly, the high rates of re-infection among patients that initially clear incident infections indicate a high background burden of disease, high HBV transmission rates as well as high rates of unprotected sex among HIV infected patients. This emphasizes the need for comprehensive prevention services within HIV care programmes that includes routine HBV vaccination to all HIV infected patients, provision of condoms coupled with, health promotion education that includes correct condom use, and risk reduction counselling to this audience. The high rate of HBV re-infection also suggests that patients with HIV co-infection on ART may not be developing sufficient protective immunity following natural exposure to HBV infection, suggesting persistently inadequate functional immunity in these patients [24, 25]. There is also growing evidence that HIV infected patients have suboptimal antibody responses to HBV vaccine in both infants and adults, with some studies recommending reinforced or accelerated vaccination in HIV co-infected patients [26, 27].

Our study found that men had a significantly higher prevalence of HBV at baseline and had a higher risk of incident as well as persistent HBV infection. This finding is in keeping with data from Asian patients, where male gender was identified as an independent negative prognosticator for chronic HBV infection and its complications [28]. HBV prevalence was also found to be more common among males than females (15.4% vs 10.1%, *p* = 0.001), within a cohort of HIV infected patients enrolled between 2004 and 2007 in Nigeria [29]. Data from an older American cohort study in patients receiving haemodialysis showed evidence of a sex difference in response to hepatitis B virus, probably explaining the greater incidence of several chronic liver diseases, including primary hepatocellular carcinoma, in males [30]. The role of gender needs to be investigated with further studies.

The presence of TB at baseline was associated with a 2-fold increased risk of incident HBV infection, suggesting that HIV, TB and HBV co-infection may drive each other creating a syndemic. Advanced immunosuppression as evidenced by CD4 count < 200 cells/mm^3^ was not associated with incident HBV infection or higher prevalence of HBV at baseline. This finding is similar to other cohorts where baseline CD4 count was not significantly different between HBV infected and HBV uninfected groups [11, 31, 32]. However, the risk of persistent HBV infection was found to be higher with severe immunosuppression, demonstrating the importance of immune-competence in clearing HBV infection. The data suggest that HIV confers a risk for HBV persistence, hence the need for targeted prevention in this high-risk group. The role of adaptive anti-HBV responses in the control of HBV has been reviewed elsewhere [33].

The limitation in our retrospective study is not analyzing the effect of different ART drug regimens on the incidence and persistence of HBV in HIV co-infected patients. All ART regimens that patients received would have included lamivudine and introduced Tenofovir in the latter part of 2013 as HBV active ART. The Ugandan study by Seremba et al, did not find any new infections in patients receiving Tenofovir based ART, while some new infections occurred with Lamivudine-only based ART [23].

Not all patients had longitudinal follow up, and the smaller number of patients in follow up is a limitation and may have resulted in the underestimation of the true burden of disease. Other testing limitations include not determining HBV surface antibodies at baseline and not testing for HBV core antibodies at any time during follow up.

With the presence of an effective vaccine against HBV, HIV and TB have overshadowed its prominence as a significant infectious disease over the past 3 decades. There is however a renewed realization that the burden of HBV continues to increase in the post-vaccination era [2].

High HBV prevalence, incidence and re-infection rates pose a serious public health threat from ongoing HBV transmission as well as from enhancing HIV-infected patients’ risk to non-AIDS comorbidities such as cirrhosis and hepatocellular carcinoma. These data highlight the vulnerability of the South African population to infectious disease syndemics that include HBV, HIV, TB, and the ongoing morbidity and mortality, associated with these conditions. These co-occurring conditions intertwined with similar biologic, social and environmental drivers, are likely to foster emergence of other epidemics in the future. An urgent appraisal of how the public health system and communities are better able to identify and respond to these epidemics, in order to mitigate their impact is warranted. We illustrate ongoing acquisition of HBV in adult patients receiving HIV care, and suggest that expanding the rollout of HBV vaccination and integrating it as part of comprehensive HIV care in countries where HBV is endemic, is the first step in a wholistic response to the HIV-HBV-TB syndemics. While widespread ART access offers promising viral suppression in HBV-HIV co-infected individuals, this strategy is not a route to global eradication of HBV and HIV. Additional investment and resources aimed at improving routine diagnostic screening while reducing the number of undiagnosed cases, development of curative therapy, and improvements in vaccine and drug coverage, would be appropriate responses to the call by the World Health Assembly, towards the global elimination of HBV as a public health threat by 2030 [34].

## Conclusion

This work demonstrated a high incidence of HBV infection in patients receiving care for HIV and TB in South Africa. We conclude that provision of HIV care and treatment in high HBV burden settings provide a missed opportunity for HBV screening, immunization and care provision.

## Data Availability

The datasets used and/or analysed during the current study are available from the corresponding author on reasonable request.

## Abbreviations

HBV: Hepatitis B virus
HBsAg: Hepatitis B surface antigen
TB: Tuberculosis
ART: Antiretroviral treatment
HIV: Human immunodeficiency virus
SD: Standard deviation
HR: Hazard-ratio
aHR: Adjusted hazard ratio
BMI: Body mass index
LTFU: Loss to follow-up
